# Design and Multi-Country Validation of Text Messages for an mHealth Intervention for Primary Prevention of Progression to Hypertension in Latin America

**DOI:** 10.2196/mhealth.3874

**Published:** 2015-02-18

**Authors:** Francisco Diez-Canseco, J Alfredo Zavala-Loayza, Andrea Beratarrechea, Rebecca Kanter, Manuel Ramirez-Zea, Adolfo Rubinstein, Homero Martinez, J Jaime Miranda

**Affiliations:** ^1^CRONICAS Center of Excellence in Chronic DiseasesUniversidad Peruana Cayetano HerediaLimaPeru; ^2^South American Center for Cardiovascular Health (CESCAS)Institute for Clinical Effectiveness and Health PolicyBuenos AiresArgentina; ^3^INCAP Research Center for the Prevention of Chronic Diseases (CIIPEC)Institute of Nutrition of Central America and PanamaCiudad de GuatemalaGuatemala; ^4^RAND CorporationSanta Monica, CAUnited States; ^5^Hospital Infantil de México Federico GómezCiudad de MéxicoMexico; ^6^School of MedicineUniversidad Peruana Cayetano HerediaLimaPeru

**Keywords:** cross-cultural comparison, developing countries, health literacy, hypertension, Latin America, mHealth, preventive medicine, prehypertension, text messages, validation studies

## Abstract

**Background:**

Mobile health (mHealth) has been posited to contribute to the reduction in health gaps and has shown fast and widespread growth in developing countries. This growth demands understanding of, and preparedness for, local cultural contexts.

**Objective:**

To describe the design and validation of text messages (short message service, SMS) that will be used for an mHealth behavioral change intervention to prevent hypertension in three Latin American countries: Argentina, Guatemala, and Peru.

**Methods:**

An initial set of 64 SMS text messages were designed to promote healthy lifestyles among individuals in different stages of behavior change, addressing four key domains: salt and sodium intake, fruit and vegetable intake, consumption of high fat and sugar foods, and physical activity. The 64 SMS text messages were organized into nine subsets for field validation. In each country 36 people were recruited, half of them being male. Of the participants, 4 per country evaluated each subset of SMS text messages, which contained between 6 and 8 SMS text messages regarding different key domains and stages of change. The understanding and appeal of each SMS text message was assessed using a 7-item questionnaire. The understanding and appeal ratings were used to reach a final set of 56 SMS text messages.

**Results:**

Overall, each of the 64 SMS text messages received a total of 12 evaluations (4 per country). The majority of evaluations—742 out of a total of 767 (96.7%) valid responses—revealed an adequate understanding of the key idea contained in the SMS text message. On a scale from 1 to 10, the average appeal score was 8.7 points, with a range of 4 to 10 points. Based on their low scores, 8 SMS text messages per country were discarded. Once the final set of 56 SMS text messages was established, and based on feedback obtained in the field, wording and content of some SMS text messages were improved. Of the final set, 9, 8, and 16 of the SMS text messages were improved based on participant evaluations from Argentina, Guatemala, and Peru, respectively. Most SMS text messages selected for the final set (49/56, 88%) were the same in all countries, except for small wording differences.

**Conclusions:**

The final set of SMS text messages produced had very high rates of understanding and appeal in three different Latin American countries. This study highlights the importance of developing and validating a package of simple, preventative SMS text messages, grounded in evidence and theory, across three different Latin American countries with active engagement of end users.

## Introduction

It has been more than a decade since multimedia, electronic communications, and networking technology were raised up as, and expected to be, useful tools to increase health literacy [1]. Health literacy has been defined as “the degree to which individuals have the capacity to obtain, process, and understand basic health information and services needed to make appropriate health decisions” [1,2]. Health literacy remains challenging in both developed and developing countries [2,3]. From a public health standpoint, this is worrisome at a global scale—individual knowledge about risk factors and behavior to prevent or ameliorate risk to poor health is key for primary prevention activities, as well as for actual health care seeking, provision, and compliance [4].

Simultaneously, the expectations on mobile health (mHealth) to reduce large health gaps have increased. The term mHealth is defined as the use of mobile and wireless devices to improve health-related outcomes, services, and research [5]. For developing countries in particular, one of the key mHealth applications used to deliver health education to patients is the use of text messaging (short message service, SMS) [6,7], yet the evidence of health improvement generated from this application is limited [8,9]. The potential advantages to contacting large numbers of laypeople or patients through SMS text messaging make this an enticing route for the delivery of health education.

However, different researchers and health care providers have realized that the use of mHealth technologies, including SMS text messaging, needs to accommodate, and be tailored to, potential literacy challenges, as it has been increasingly obvious that health literacy is not only about reading [10]. In addition to this, mHealth technologies have experienced fast and wide geographical spread. This demands understanding of local cultural contexts, as well as the adaptation of contents and strategies, to guarantee adequate understanding and appeal of mHealth innovations by end users. The objective of this study is to describe the process and results of the development and validation of SMS text messages to be used in an mHealth behavioral change intervention for the primary prevention of hypertension in three Latin American countries: Argentina, Guatemala, and Peru.

## Methods

### The Problem: Progression to Hypertension

In developing countries, hypertension is the leading risk factor contributing to global disease burden [11]. Prehypertension requires no medical treatment and is defined as the condition where an individual has systolic blood pressure values in the 120 to 139 mmHg range or diastolic blood pressure in the 80 to 89 mmHg range [12]. However, the rate of progression to hypertension is relatively high—10 to 20% per year [13]. Lowering systolic blood pressure by as little as 2 mmHg in a prehypertensive individual, before the appearance of clinical hypertension, could have a significant impact on lowering vascular mortality—about 10% lower stroke mortality and about 7% lower mortality from ischemic heart disease or other vascular causes in middle age [14]. The prevalence of prehypertension among the adult population in Latin American urban settings is between 22% and 35% [15,16]. However, not all prehypertensive subjects become hypertensive individuals, as this condition can be prevented through the adoption of healthy lifestyles and without the use of medication [12,17].

### Description of the mHealth Intervention

The design and validation of SMS text messages was one of the components of the larger trial “Use of Mobile Technology to Prevent Progression of Pre-hypertension in Latin American Urban Settings” (ClinicalTrials.gov NCT01295216). The main objective of this trial is to evaluate the effectiveness of an intervention to reduce blood pressure and prevent progression to hypertension in prehypertensive individuals living in low-income urban settings in Argentina, Guatemala, and Peru. The primary hypothesis of this trial is that prehypertensive subjects who receive mHealth support for 12 months (intervention group) will have lower blood pressure compared to individuals who receive the usual primary health care (control group).

The mHealth intervention included monthly mobile phone calls and weekly SMS text messages to promote lifestyle modification that may help to reduce blood pressure. The mobile phone calls were developed according to the motivational interview technique [18,19] and tailored to the individual stage of change [20]. Each phone call, delivered by trained nutritionists, focused on a specific domain and was followed by 4 to 5 domain-specific text messages, delivered once per week.

The contents of the mHealth intervention were developed to improve lifestyle habits in four key domains related to hypertension prevention: salt and sodium intake, fruit and vegetable intake, consumption of high fat and sugar foods, and physical activity. The manual developed for community health workers by the National Heart, Lung, and Blood Institute, “Su Corazón, Su Vida. Manual del Promotor y Promotora de Salud” (Your Heart, Your Life) served as the basis to inform the four selected domains [21]. Activities described in this manual were complemented by other recommendations and were specifically reviewed to support the content of the text messages designed for the mHealth intervention [22,23].

### Development of SMS Text Messages

Development of the SMS text messages content was based on the Transtheoretical Model, also known as the Stages of Change Model. This model proposes five stages toward behavior change: precontemplation, contemplation, preparation, action, and maintenance [24,25]. For this intervention, these five stages were grouped into three stages. A detailed description of the type of content and number of text messages required per stage of change is shown in Table 1.

Each of the four key domains required a minimum of 14 text messages, for a final set of 56 text messages. In working toward this aim, we designed and tested 64 text messages for all four domains, assuming that some would not be as well understood as others. Of the 64 text messages, 6 were targeted to men only (eg, addressing situations such as eating outside the home), 6 were for women only (eg, focused around cooking practices), and the remaining 52 were targeted at both men and women.

**Table 1:** Stages of change, type of content, and number of SMS text messages per key domain.

Stage of change	Type of content of SMS text message	Example of SMS text message^a^	Number of SMS text messages per key domain	
			Developed for validation	Required for intervention
Stage 1: precontemplation and contemplation	Information about the benefits of developing healthy lifestyles.	If you use just a small amount of salt when cooking, you are helping your family to keep healthy blood pressure.	6	5
Stage 2: preparation and action	Tips and suggestions for behavioral change toward healthy lifestyles.	Cook your own lunch to take to work. This way you can use less salt and make it healthier than sandwiches and fast food.	6	5
Stage 3: maintenance	Positive reinforcement of achievements in healthy lifestyles.	Keep protecting your heart. Eat less than a teaspoon of salt each day.	4^b^	4^b^
Total text messages per key domain			16	14
Total text messages for all four key domains			64	56

^a^All examples provided are text messages from the salt and sodium intake key domain.

^b^The variety of content amenable to reinforcement by text messages was much more limited for stage 3 than for the other two stages. Thus, to avoid repeating content, only 4 text messages were required in this stage of change. During validation these text messages were not discarded, but reworded to make their content clearer.

For each key domain, the design of the text message content was defined according to some specific topics or behaviors (see Table 2). For example, the text message within the domain *salt and sodium intake* included the following topics: reduction in the use of salt when cooking and at the table, replacement of salt with other spices, reduced consumption of salty foods, and benefits of sodium reduction on cardiovascular health and blood pressure.

**Table 2:** Selected key domains and examples of specific topics prioritized in the design of SMS text messages.

Key domain	Examples of specific topics prioritized
Salt and sodium intake	Reduction in the use of salt when cooking and at the table. Replacement of salt with other spices such as oregano, pepper, and chili. Reduction or avoidance of deli meat, fast food, chips, snacks, and other salty foods. Replacement of deli meat sandwiches with nonprocessed meat and vegetable sandwiches. Benefits to blood pressure and cardiovascular health.
Fruit and vegetable intake	Replacement of snacks with fruits and vegetables. Benefits of buying seasonal fruits and vegetables. Benefits to blood pressure and cardiovascular health. Benefits to body weight and physical appearance.
Consumption of high fat and sugar foods	Reduction or avoidance of soft drinks, sweets, and added sugar. Reduction or avoidance of deli meat, margarine, chips, and other fatty foods. Replacement of saturated fats with unsaturated ones. Benefits to blood pressure and cardiovascular health. Benefits to body weight and physical appearance.
Physical activity	Increase of activity in daily routines. Involvement in group activities. Benefits to physical and mental health. Benefits to body weight and physical appearance.

All 64 SMS text messages met the following criteria: maximum length of 140 characters, preferably only one (in some cases, up to two) concepts covered, and the use of simple and direct language, understandable by laypeople from poor Latin American urban settings.

Before reaching the field validation stage, all 64 text messages were reviewed in each country by local communicators to assure adequacy of language, since there are regional variations around common words or phrases. This yielded three country-specific sets of 64 text messages that then underwent a validation process in each country.

### Validation of SMS Text Messages

#### Overview

In this section we describe the guiding principles for the SMS text message validation, its design, and methodology, as well as the analysis approach.

#### Guiding Principles

The following guiding principles were agreed upon beforehand between participating countries: (1) the process would accommodate local timelines and resources, (2) the validation process would be carried out for each SMS text message, and (3) the final set of text messages could differ between countries.

During the validation, two main areas were to be prioritized: SMS text message understanding and appeal. This process would lead to the elimination of some text messages and to improvements in the wording of others.

#### Design and Methodology

All 64 SMS text messages were grouped into nine subsets for validation, with 6 to 8 text messages in each subset. As planned, some of the topics would be gender oriented, so one of the subsets had 6 text messages targeted to women and another subset had 6 text messages targeted to men. The remaining seven subsets had mixed text messages that were not gender specific. Each subset had a combination of text messages covering different key domains and stages of change to guarantee that participants—with unknown status of their stages of change across the four key domains—had a chance to comment on a variety of text messages. An example of one text message subset used during the validation process is provided in Table 3.

**Table 3:** Example of a subset of SMS text messages used for validation (set 3, Peru).

SMS text message number	Key domain	Stage of change	SMS text message used in testing	
			Spanish ^a^	English
1	Salt and sodium intake	Stage 2: preparation and action	Prueba preparar tu sanguche con tomate, lechuga, y pollo en lugar de usar embutidos como jamon o salchichas, que tienen mucha sal.	Try and use chicken and fresh vegetables, such as tomato and lettuce, instead of deli meat for your sandwiches. Deli meat is high in salt.
2	Fruit and vegetable intake	Stage 1: precontemplation and contemplation	Frutas como platanos, ciruelas, melones, melocotones, y naranjas te ayudan a tener la presion normal.	Fruits, such as bananas, plums, melons, peaches, and oranges, help you keep normal blood pressure.
3	Fruit and vegetable intake	Stage 3: maintenance	Sigue asi. Las frutas y verduras no tienen grasa, por lo que te ayudan a bajar de peso y a verte bien.	Keep it up. Fruits and vegetables are fat free—they help you lose weight and look good.
4	Consumption of high fat and sugar foods	Stage 1: precontemplation and contemplation	Si comes menos manteca, visceras, salchichas, jamon, y otros embutidos, podras bajar tu colesterol y tu presión.	Eating less animal fat, liver, heart, kidneys, gizzard, sausage, ham, and other deli meat helps lower your cholesterol and blood pressure.
5	Consumption of high fat and sugar foods	Stage 2: preparation and action	Para comer menos grasa, quítale el pellejo al pollo y la grasa a la carne antes de cocinarla o comerla.	To eat less fat, remove skin from chicken and fat from meat before you cook or eat it.
6	Physical activity	Stage 1: precontemplation and contemplation	Haciendo actividad física al menos 30 minutos al día podrás bajar tu presión y evitar enfermedades.	Doing physical activity at least 30 minutes per day can lower your blood pressure and prevent diseases.
7	Physical activity	Stage 2: preparation and action	Si tienes que ir cerca de tu casa, no tomes combi o mototaxi.^b^¡Es mejor caminar! Así no gastas en pasaje y haces actividad física.	If you are not going far, avoid riding a bus or mototaxi.^b^It’s better to walk! It’s cheaper and allows you to do some physical activity.

^a^Peruvian wording. SMS text messages were written without accents to avoid having mobile phones replace them with other characters.

^b^Mototaxis are a type of Peruvian rickshaw widely used in shanty towns.

A 7-item questionnaire with open- and closed-ended questions was designed for the assessment of each SMS text message. The questionnaire included 3 questions about the understanding of SMS text messages, 3 questions about text message appeal, and 1 final question requesting suggestions on how to improve the text messages (see Multimedia Appendix 1). Trained interviewers administered the questionnaires in face-to-face interactions and registered the participants’ answers in writing.

Understanding was evaluated by asking each person to describe the content of the message as if she or he was explaining it to another person. If the core message was communicated, the message was rated as being understood. If the core message was not communicated, the message was rated as not being understood. Additionally, interviewees were asked whether there was any word whose meaning was not understood.

Appeal was rated on a *dislike-like* point scale from 1 to 10, asking interviewees to assign a numerical rating from low (1, *disliked the most*) to high (10, *liked the most*) according to how much they liked or disliked the message.

Validation instruments were first tested in Peru in August 2011. Following this, instruments were revised and improved, and all three countries conducted the validation study in October 2011.

### Setting and Participants

Consistent with the larger trial that would utilize the SMS text messages, the validation was conducted in low-income, urban Spanish-speaking communities in or around the capital city of Argentina, Guatemala, and Peru.

In each country, 4 people evaluated each of the nine SMS text message subsets. In all countries, 108 participants (36 per country) were recruited for a one-on-one, 30-minute interview. Participants were aged between 30 and 60 years old, equally divided by gender, could read, and owned a mobile phone. The gender-specific text message subsets were evaluated either by women or men, accordingly.

### Analysis of Validation

The analysis of each SMS text message was focused around two main areas: understanding of the content and an appeal score. Questions used in the validation process are provided in Multimedia Appendix 1.

The assessment of text message understanding was performed by two reviewers (FDC, JAZ) as in the following four steps.

1. Reviewers jointly examined all the written descriptions provided by participants and agreed on whether they revealed an understanding of the text message’s key message or not.

2. For those text messages where participants reported a lack of understanding, or where judges agreed that the participant did not understand the text message, the message was checked for wording and grammar.

3. Specific words that did not appear to be understood or were disliked by participants were identified.

4. The content and wording of the text messages were improved by taking into account the comments and suggestions made by the participants.

To evaluate appeal, in every country, 4 participants ranked each text message on a point scale from 1 (*disliked the most*) to 10 (*liked the most*). These four scores were averaged for each country, and an appeal score per text message was used to rank the messages within the same stage of the same key domain. For example, in each country, the 6 text messages developed for stage 1 in the physical activity domain were ranked based on their average appeal score. This ranking by domain, stage of change, and country was needed to identify the lowest-scoring text messages to be potentially discarded (see Multimedia Appendix 2).

The 2 text messages with the lowest scores were reviewed again. Priority was given to the understanding of the messages’ key ideas, and taking into account the participants’ opinions and suggestions about each message. After this exercise, 1 of the 2 messages was discarded by consensus between reviewers.

### Ethics

The study protocol, field instruments, and informed consent forms were reviewed and approved by local Institutional Review Boards (IRB) in Argentina, Guatemala, and Peru, as well as RAND Corporation’s IRB. All participants were informed about the study and gave oral consent.

## Results

### Overview

A total of 108 participants (36 per country) took part in the study. The average age was 42 (SD 8.5) years old and 50% (54/108) were male. We expected to collect four evaluations for each of the 64 text messages in every country, with a target of 768 total evaluations. One evaluation was missing at the end of the validation study, thus, a total of 767 valid evaluations were analyzed.

### Understanding of SMS Text Messages

According to the judges, 96.7% (742/767) of the evaluations revealed an adequate understanding of the key idea contained in the text message. This figure was very similar in Argentina (248/255, 97.3%), Guatemala (247/256, 96.5%), and Peru (247/256, 96.5%). Of all the responses, feedback provided by Argentinian participants tended to be more detailed and clearer than that from Guatemala and Peru.

Across countries, only 4.0% of answers (31/767) indicated that participants did not understand the text message, or at least one word in it (8/255, 3.1% in Argentina, 8/256, 3.1% in Guatemala, 15/256, 5.9% in Peru). Examples of words or expressions that were not understood by some respondents included nutrition- and health-related words, such as “fibras” (fiber, in Peru), “frutas de estación” (seasonal fruits, in Argentina), and “físicamente activo" (physically active, in all three countries).

### Appeal of SMS Text Messages

The appeal scores for text messages ranged from 4 to 10 points. The average score for all 64 text messages in all three countries was 8.7 (SD 1.8) points. The average scores for Guatemala, Argentina, and Peru were 9.4 (SD 1.4), 8.4 (SD 1.5), and 8.3 (SD 2.2), respectively. Detailed scoring per country, key domain, and stage of change are shown in Table 4.

In Guatemala, 19 out of 64 (30%) SMS text messages received the highest average score of 10 points. Only 2 out of 64 (3%) text messages in Argentina and 0 out of 64 (0%) messages in Peru received a score of 10. Within each domain and stage of change subset, the lowest-ranked text messages were not always the same across countries, although some coincidences were noted. Detailed scoring per text message by country is provided in Multimedia Appendix 2.

In all three countries, 1 text message that did not appeal to some participants was “¿Por qué no haces algo distinto? Empezar tu día con una fruta te ayudará a estar sano y no engordar,” which translates into English as “Why not try something different? Starting your day with fruit will help you be healthy and avoid getting fat." This message was unappealing to some participants because “getting fat” was considered slightly hurtful by some people. Also, 1 text message in particular that was criticized across all countries was “Cuéntales a las mujeres de tu hogar que las frutas y las verduras ayudan a bajar el colesterol y protegen el corazón,” which translates into English as “Tell the women in your household that fruits and vegetables help lower cholesterol levels and protect the heart.” The latter was a male-directed text message with instructions targeting the women of the household. Therefore, participants considered this information to be not only relevant for communicating to females, but for all the family members.

**Table 4:** SMS average appeal scores by country, key domain, and stage of change.

Key domain and stage of change		SMS text message appeal score, mean (SD)			
		Argentina	Guatemala	Peru	Total
**Salt and sodium intake**					
	All stages	8.13 (1.54)	9.16 (1.60)	8.30 (2.11)	8.53 (1.81)
	Precontemplation and contemplation	7.83 (1.61)	8.92 (1.84)	8.42 (1.72)	8.39 (1.76)
	Preparation and action	8.25 (1.51)	9.33 (1.55)	7.79 (2.45)	8.46 (1.97)
	Maintenance	8.38 (1.50)	9.25 (1.29)	8.88 (2.03)	8.83 (1.64)
**Fruit and vegetable intake**					
	All stages	8.42 (1.50)	9.44 (1.51)	8.64 (1.67)	8.84 (1.62)
	Precontemplation and contemplation	8.58 (1.53)	9.38 (1.44)	8.75 (1.15)	8.90 (1.41)
	Preparation and action	8.41 (1.33)	9.29 (1.99)	8.42 (2.15)	8.71 (1.89)
	Maintenance	8.19 (1.72)	9.75 (0.45)	8.81 (1.60)	8.92 (1.50)
**Consumption of high fat and sugar foods**					
	All stages	8.27 (1.73)	9.28 (1.33)	7.80 (2.75)	8.45 (2.11)
	Precontemplation and contemplation	8.54 (1.64)	9.25 (1.26)	7.96 (2.61)	8.58 (1.97)
	Preparation and action	7.63 (2.02)	9.46 (1.25)	7.67 (2.87)	8.25 (2.29)
	Maintenance	8.81 (1.05)	9.06 (1.57)	7.75 (2.93)	8.54 (2.05)
**Physical activity**					
	All stages	8.76 (1.35)	9.59 (1.03)	8.56 (1.98)	8.97 (1.56)
	Precontemplation and contemplation	9.00 (1.21)	9.63 (0.97)	7.83 (2.62)	8.82 (1.89)
	Preparation and action	8.43 (1.65)	9.54 (0.98)	8.75 (1.51)	8.92 (1.46)
	Maintenance	8.88 (1.02)	9.63 (1.26)	9.38 (0.89)	9.29 (1.09)
Total (all domains and stages)		8.39 (1.55)	9.37 (1.39)	8.32 (2.17)	8.70 (1.80)

### Selection and Improvement of the Final Set of SMS Text Messages

As per the protocol to provide enough variation per week throughout a year, the final SMS text message set was planned to include 56 messages in each country—6 targeted to men, 6 targeted to women, and 44 for both genders. As mentioned in the methodology section, of all the text messages tested within each domain and stages of change 1 and 2, a total of 8 messages were discarded. Finally, to assemble the final set, some of the remaining 56 messages were modified to improve understanding. Out of a total 56 messages per country, 16 were modified for Peru, 9 were modified for Argentina, and 8 were modified for Guatemala.

The text message selection followed the same process in all three countries and, as anticipated, most messages selected for the final set (49/56, 88%) were the same except for small wording differences. The final text message sets can be seen in Multimedia Appendix 3.

## Discussion

### Principal Findings

A standardized process for the design and validation of SMS text messages, a key component of an mHealth intervention for the prevention of hypertension, was developed. Through this methodology we were able to obtain three country-specific sets of 56 text messages with high levels of appeal and understanding among individuals from low-income, peri-urban communities. This process and its product, the final set of text messages, were designed to accomplish an ambitious and complex challenge—to promote behavioral change among nonsymptomatic individuals at risk of developing hypertension. In so doing, it embraced the following: (1) the tackling of four behaviors—salt and sodium intake, fruit and vegetable intake, consumption of high fat and sugar foods, and physical activity, (2) the discrimination between three distinct stages of change within each behavior, and (3) being mindful of gender differences.

As text messaging interventions become more common, it is necessary to make the procedures behind their development explicit [26]. In this scenario, our study yielded a number of valuable lessons related to the design and validation of text messages: (1) application of theoretical frameworks in the behavioral change and health communication domains, (2) evidence-based selection of lifestyle habits to target, (3) language and cultural adaptation of text message content, and (4) involvement of end users in message validation. These four aspects, further detailed below, were devised for the implementation of a prevention strategy for hypertension, but can be applied effectively to other conditions.

First, regarding theoretical frameworks, it has been postulated that “if SMS (text message) intervention studies are built on evidence and theory, the potential impact of these studies will be much greater” [27]. In our case, the use of the Transtheoretical Model [24,25] signaled the need to develop SMS text messages that were tailored to specific stages of change, and guided the phrasing of initial versions of the messages. This procedure was further supported by health communications frameworks [28] that recommend the use of “gain-framed messages” [29], which emphasize the benefits of taking action, when developing initiatives to promote behaviors that prevent the onset of disease.

Second, reviewing available evidence on hypertension prevention was useful on two fronts. Initially, this approach helped in selecting the four lifestyles to focus on. For example, reducing tobacco and alcohol use were excluded during the initial planning phases due to their smaller effects on lowering blood pressure. Then, this evidence-based approach was also helpful in prioritizing actions and recommendations within each habit to be delivered via text message.

Third, being aware that social context influences behavioral change and that “communication is not simply message repetition” [30], we placed special attention onto the cultural and language adaptation of the text message content. This was done in all three countries and involved researchers, health communicators, and end users. The tailoring of health messages and counseling strategies to be sensitive to the cultural beliefs, values, language, literacy, and customs of the target population has been labeled with the strongest level of evidence [31].

Fourth, as depicted by the quote “focus on the user and all else will follow” [32], our validation process incorporated an engagement phase with end users. Their evaluations and opinions were a major contribution to defining the final set of text messages.

We believe that our validation design is strong as it brought together aspects of theory, evidence, and context to develop and validate three sets of 56 SMS text messages across Latin American settings. Our text messages had a 97% level of understanding and an average of 8.7 out of 10 points in appeal, thus they could be useful beyond the original study for which they were developed. Noticeably, in all countries the understanding and appeal of the text message contents were quite high, when on an *a priori* scenario, we could have expected more differences given the diversity of contexts. In this sense, the absence of major differences in understanding or appeal between the three countries or by gender reflect, importantly, that the linguistic and cultural adaptation conveyed during the development process of the study was adequate at all sites. When subjects elaborated in their own words about understanding, however, there were some slight differences by country. Argentinian subjects tended to give more extended or detailed responses when asked about understanding, which may be a reflection of the higher average level of education in Argentina compared to Guatemala or Peru. There were also slight country-specific differences in terms of appeal. Guatemalans tended to be kinder in their ratings, as shown by their higher average ratings compared to the other sites. For example, 19 text messages received the maximum of 10 points in Guatemala, compared to only 2 in Argentina and 0 in Peru. Yet, overall understanding and appeal were not markedly different across countries or by gender and, thus, pose no threat to the validity of our findings.

###  Limitations

Any validation study requires striking a balance between depth and breadth of the exercise, together with its matching resources. Our study introduced, to its strength, a combination of multi-country sites, four key behavioral domains, and three behavioral stages of change aiming to achieve a final set of 56 SMS text messages to be used weekly during a 12-month period. The resources available, in terms of project’s time allocation and financial means, to accomplish such a considerable goal, only allowed 4 people per country to review each text message. Despite the limited number of evaluators per individual message, this process was systematically repeated for all 64 messages, resulting in high levels of understanding and appeal. Future studies should find the right balance between the number of text messages to be developed per domain, stage of change, and gender to determine the optimal sample size needed for subsequent validation studies.

An additional minor limitation was that we did not have the means to audiotape the answers provided, and having interviewers’ handwritten notes may have introduced some observer bias or resulted in missing information that was not recorded. Despite this limitation, overall, the registries were considered by the research team to be very good. To assess text message appeal, we used a short focused instrument with only 7 items for each message to avoid introducing unnecessary complications to the participant’s assessment of the message. We acknowledge that other instruments may be able to capture appeal features in greater detail. Again, a balance between the size of the task and available resources will clearly impact such decisions regarding which instrument to use. Future studies could have greater focus on tailoring the text messages to actual transitions between stages of change, be it on the direction of change, or in the number of behaviors that demonstrate actual change. As this was not part of our study design, we did not consider these temporal changes in designing our text messages.

### Conclusions

In today’s global health context, mHealth has secured a place as a dynamic field with the potential to reduce health gaps and disparities by improving health behaviors and disease self-management, as well as facilitating health care delivery processes [33,34]. As with any evolving area, more emphasis on process and methodology, such as those described in this validation study, is required. Taking into consideration that SMS text messaging is one of many delivery channels, clarity of the communication is key [2], and very few studies have properly described their methods and criteria used for the development and validation of the content of text messages [26,35,36].

This study highlights the importance of developing and validating a package of simple, preventative SMS text messages, grounded in evidence and theory, across three different Latin American countries with active engagement of end users. We anticipate that the potential impact of these text message sets will be much greater—they could serve as a resource for several different mHealth approaches geared toward improving lifestyle-related behaviors for the prevention of hypertension and other related chronic noncommunicable diseases.

## Multimedia Appendix 1

SMS text message validation questionnaire.

## Multimedia Appendix 2

Ranking of SMS text messages per country.

## Multimedia Appendix 3

Final sets of 56 SMS text messages per country.

## Abbreviations

IRB: Institutional Review Board
SMS: short message service

## References

[1] Selden C, Zorn M, Ratzan SC, Parker RM (2000). US National Library of Medicine, NIH.

[2] Nielsen-Bohlman L, Panzer AM, Kindig DA (2004). Health Literacy: A Prescription to End Confusion.

[3] Nutbeam D (2000). Health literacy as a public health goal: a challenge for contemporary health education and communication strategies into the 21st century. Health Promot Int.

[4] Parker RM, Ratzan SC, Lurie N (2003). Health literacy: a policy challenge for advancing high-quality health care. Health Aff (Millwood).

[5] Mechael PN (2009). The case for mHealth in developing countries. Innovations: Technology, Governance, Globalization.

[6] Déglise C, Suggs LS, Odermatt P (2012). Short message service (SMS) applications for disease prevention in developing countries. J Med Internet Res.

[7] Déglise C, Suggs LS, Odermatt P (2012). SMS for disease control in developing countries: a systematic review of mobile health applications. J Telemed Telecare.

[8] Beratarrechea A, Lee AG, Willner JM, Jahangir E, Ciapponi A, Rubinstein A (2014). The impact of mobile health interventions on chronic disease outcomes in developing countries: a systematic review. Telemed J E Health.

[9] Fjeldsoe BS, Marshall AL, Miller YD (2009). Behavior change interventions delivered by mobile telephone short-message service. Am J Prev Med.

[10] Schonlau M, Martin L, Haas A, Derose KP, Rudd R (2011). Patients' literacy skills: more than just reading ability. J Health Commun.

[11] Lim SS, Vos T, Flaxman AD, Danaei G, Shibuya K, Adair-Rohani H, Amann M, Anderson HR, Andrews KG, Aryee M, Atkinson C, Bacchus LJ, Bahalim AN, Balakrishnan K, Balmes J, Barker-Collo S, Baxter A, Bell ML, Blore JD, Blyth F, Bonner C, Borges G, Bourne R, Boussinesq M, Brauer M, Brooks P, Bruce NG, Brunekreef B, Bryan-Hancock C, Bucello C, Buchbinder R, Bull F, Burnett RT, Byers TE, Calabria B, Carapetis J, Carnahan E, Chafe Z, Charlson F, Chen H, Chen JS, Cheng AT, Child JC, Cohen A, Colson KE, Cowie BC, Darby S, Darling S, Davis A, Degenhardt L, Dentener F, Des Jarlais DC, Devries K, Dherani M, Ding EL, Dorsey ER, Driscoll T, Edmond K, Ali SE, Engell RE, Erwin PJ, Fahimi S, Falder G, Farzadfar F, Ferrari A, Finucane MM, Flaxman S, Fowkes FG, Freedman G, Freeman MK, Gakidou E, Ghosh S, Giovannucci E, Gmel G, Graham K, Grainger R, Grant B, Gunnell D, Gutierrez HR, Hall W, Hoek HW, Hogan A, Hosgood HD, Hoy D, Hu H, Hubbell BJ, Hutchings SJ, Ibeanusi SE, Jacklyn GL, Jasrasaria R, Jonas JB, Kan H, Kanis JA, Kassebaum N, Kawakami N, Khang YH, Khatibzadeh S, Khoo JP, Kok C, Laden F, Lalloo R, Lan Q, Lathlean T, Leasher JL, Leigh J, Li Y, Lin JK, Lipshultz SE, London S, Lozano R, Lu Y, Mak J, Malekzadeh R, Mallinger L, Marcenes W, March L, Marks R, Martin R, McGale P, McGrath J, Mehta S, Mensah GA, Merriman TR, Micha R, Michaud C, Mishra V, Mohd Hanafiah K, Mokdad AA, Morawska L, Mozaffarian D, Murphy T, Naghavi M, Neal B, Nelson PK, Nolla JM, Norman R, Olives C, Omer SB, Orchard J, Osborne R, Ostro B, Page A, Pandey KD, Parry CD, Passmore E, Patra J, Pearce N, Pelizzari PM, Petzold M, Phillips MR, Pope D, Pope CA, Powles J, Rao M, Razavi H, Rehfuess EA, Rehm JT, Ritz B, Rivara FP, Roberts T, Robinson C, Rodriguez-Portales JA, Romieu I, Room R, Rosenfeld LC, Roy A, Rushton L, Salomon JA, Sampson U, Sanchez-Riera L, Sanman E, Sapkota A, Seedat S, Shi P, Shield K, Shivakoti R, Singh GM, Sleet DA, Smith E, Smith KR, Stapelberg NJ, Steenland K, Stöckl H, Stovner LJ, Straif K, Straney L, Thurston GD, Tran JH, Van Dingenen R, van Donkelaar A, Veerman JL, Vijayakumar L, Weintraub R, Weissman MM, White RA, Whiteford H, Wiersma ST, Wilkinson JD, Williams HC, Williams W, Wilson N, Woolf AD, Yip P, Zielinski JM, Lopez AD, Murray CJ, Ezzati M, AlMazroa MA, Memish ZA (2012). A comparative risk assessment of burden of disease and injury attributable to 67 risk factors and risk factor clusters in 21 regions, 1990-2010: a systematic analysis for the Global Burden of Disease Study 2010. Lancet.

[12] Chobanian AV, Bakris GL, Black HR, Cushman WC, Green LA, Izzo JL, Jones DW, Materson BJ, Oparil S, Wright JT, Roccella EJ, National Heart‚ Lung‚Blood Institute Joint National Committee on Prevention‚ Detection‚ Evaluation‚Treatment of High Blood Pressure, National High Blood Pressure Education Program Coordinating Committee (2003). The Seventh Report of the Joint National Committee on Prevention, Detection, Evaluation, and Treatment of High Blood Pressure: the JNC 7 report. JAMA.

[13] Vasan RS, Larson MG, Leip EP, Kannel WB, Levy D (2001). Assessment of frequency of progression to hypertension in non-hypertensive participants in the Framingham Heart Study: a cohort study. Lancet.

[14] Lewington S, Clarke R, Qizilbash N, Peto R, Collins R, Prospective Studies Collaboration (2002). Age-specific relevance of usual blood pressure to vascular mortality: a meta-analysis of individual data for one million adults in 61 prospective studies. Lancet.

[15] Hernández-Hernández R, Silva H, Velasco M, Pellegrini F, Macchia A, Escobedo J, Vinueza R, Schargrodsky H, Champagne B, Pramparo P, Wilson E, CARMELA Study Investigators (2010). Hypertension in seven Latin American cities: the Cardiovascular Risk Factor Multiple Evaluation in Latin America (CARMELA) study. J Hypertens.

[16] Orellana-Pontaza P, Ramirez-Zea M, Barcelo A, Gil E, Gregg E, Meiners M, Valdez R, Perez-Flores E, Cafiero E (2007). Central America Diabetes Initiative (CAMDI): Survey of Diabetes, Hypertension, and Chronic Disease Risk Factors. Villa Nueva, Guatemala 2006.

[17] Ebrahim S, Smith GD (1997). Systematic review of randomised controlled trials of multiple risk factor interventions for preventing coronary heart disease. BMJ.

[18] Rollnick S, Miller WR (1995). What is motivational interviewing?. Behav Cogn Psychother.

[19] Sim MG, Wain T, Khong E (2009). Influencing behaviour change in general practice - Part 1 - brief intervention and motivational interviewing. Aust Fam Physician.

[20] Briscoe C, Aboud F (2012). Behaviour change communication targeting four health behaviours in developing countries: a review of change techniques. Soc Sci Med.

[21] National Heart, Lung, and Blood Institute (2000). Su Corazón Su Vida: Manual del Promotor y Promotora de Salud.

[22] (2008). 2008 Physical Activity Guidelines for Americans: Be Active, Healthy, and Happy!.

[23] (2010). Global Recommendations on Physical Activity for Health.

[24] US Department of Health and Human Services (1996). Physical Activity and Health: A Report of the Surgeon General.

[25] (1996). Behavior Change- A Summary of Four Major Theories: Health Belief Model, AIDS Risk Reduction Model, Stages of Change, Theory of Reasoned Action.

[26] Ybarra ML, Holtrop JS, Bağci Bosi AT, Emri S (2012). Design considerations in developing a text messaging program aimed at smoking cessation. J Med Internet Res.

[27] Cole-Lewis H, Kershaw T (2010). Text messaging as a tool for behavior change in disease prevention and management. Epidemiol Rev.

[28] Rothman AJ (2004). Is there nothing more practical than a good theory?: Why innovations and advances in health behavior change will arise if interventions are used to test and refine theory. Int J Behav Nutr Phys Act.

[29] Rothman AJ, Bartels RD, Wlaschin J, Salovey P (2006). The strategic use of gain- and loss-framed messages to promote healthy behavior: How theory can inform practice. J Commun.

[30] Ratzan SC (2001). Health literacy: communication for the public good. Health Promot Int.

[31] Artinian NT, Fletcher GF, Mozaffarian D, Kris-Etherton P, Van Horn L, Lichtenstein AH, Kumanyika S, Kraus WE, Fleg JL, Redeker NS, Meininger JC, Banks J, Stuart-Shor EM, Fletcher BJ, Miller TD, Hughes S, Braun LT, Kopin LA, Berra K, Hayman LL, Ewing LJ, Ades PA, Durstine JL, Houston-Miller N, Burke LE, American Heart Association Prevention Committee of the Council on Cardiovascular Nursing (2010). Interventions to promote physical activity and dietary lifestyle changes for cardiovascular risk factor reduction in adults: a scientific statement from the American Heart Association. Circulation.

[32] Jones SP, Patel V, Saxena S, Radcliffe N, Ali Al-Marri S, Darzi A (2014). How Google's ten Things We Know To Be True could guide the development of mental health mobile apps. Health Aff (Millwood).

[33] Free C, Phillips G, Galli L, Watson L, Felix L, Edwards P, Patel V, Haines A (2013). The effectiveness of mobile-health technology-based health behaviour change or disease management interventions for health care consumers: a systematic review. PLoS Med.

[34] Free C, Phillips G, Watson L, Galli L, Felix L, Edwards P, Patel V, Haines A (2013). The effectiveness of mobile-health technologies to improve health care service delivery processes: a systematic review and meta-analysis. PLoS Med.

[35] Irvine L, Falconer DW, Jones C, Ricketts IW, Williams B, Crombie IK (2012). Can text messages reach the parts other process measures cannot reach: an evaluation of a behavior change intervention delivered by mobile phone?. PLoS One.

[36] Shaw RJ, Bosworth HB, Hess JC, Silva SG, Lipkus IM, Davis LL, Johnson CM (2013). Development of a theoretically driven mHealth text messaging application for sustaining recent weight loss. JMIR Mhealth Uhealth.

