# Supplementary material for: Design and Multi-Country Validation of Text Messages for an mHealth Intervention for Primary Prevention of Progression to Hypertension in Latin America
Source: JMIR Mhealth Uhealth. 2015 Feb 18;3(1):e19. doi: 10.2196/mhealth.3874 (PMC4376187; doi:10.2196/mhealth.3874)
Supplement: Supplementary file 1 [file mhealth_v3i1e19_app1.pdf]

SMS set (1-9): [ ]

SMS Validation Questionnaire

ID: _________________

Country: ( ) Guatemala ( ) Argentina ( ) Peru

Gender: ( ) Male ( ) Female

Age: ____ ____ years

Date: ____ / ____ / ________

SMS № ____
(SMS content goes here)

1. If you had to explain what this SMS says, what would you say? *(Write down the exact answer)*

2. Do you understand every word in this SMS? *(If the answer is yes, skip to question 4)*

3. Which word(s) do you not understand? *(If you believe the participant doesn't understand a specific word, ask: 'what does _____ mean?')*

4. Imagine you were sent this SMS to help you improve your health. From a scale of 1 to 10, where 1 is 'I don't like this message' and 10 is 'I really like this message', what score would you give this SMS?

5. Would you please explain to me what you like about this SMS? *(Write down the exact answer)*

6. Would you please explain to me what you don't like about this SMS? *(Write down the exact answer)*

7. Do you have any suggestions on how to improve the content of this SMS? (e.g. make it shorter, longer, change a specific word or replace it for another)

Observations:
