# Supplementary material for: Design and Multi-Country Validation of Text Messages for an mHealth Intervention for Primary Prevention of Progression to Hypertension in Latin America
Source: JMIR Mhealth Uhealth. 2015 Feb 18;3(1):e19. doi: 10.2196/mhealth.3874 (PMC4376187; doi:10.2196/mhealth.3874)
Supplement: Supplementary file 2 [file mhealth_v3i1e19_app2.pdf]

ARGENTINA - SMS Appeal Ranking: Average scores by domain and stage of change (1/4)

Domain	Stage of change	Text Message (SMS)	SMS ID	Average score
Salt and sodium intake	Precontemplation & contemplation	Si preparas alimentos con poca sal ayudaras a tu familia a tener la presion normal.	1	9.25
		Podes tener tu presion normal si usas menos sal en tu comida. Protege tu corazon.	3	9.00
		Para mantener tu presion normal evita comer hamburguesas, pizza, panchos, papas fritas y manies. Tienen muchisima sal.	2	8.00
		Una persona solo necesita media cucharadita de sal al dia, pero la mayoria de gente come mas ¿Cuanto comes vos? Pensalo!	5	7.00
		Si sacas el salero de la mesa ayudaras a tu familia a comer con menos sal.	6	7.00
		Los calditos y las sopas instantaneas tienen mucha sal. Trata de evitarlos.	4	6.75
	Preparation & action	Reduce poco a poco la sal en las comidas que cocines. Asi vos y tu familia se iran acostumbrando.	7	9.25
		Proba cambiar la sal del salero por pimienta, oregano o aji.	9	8.50
		Pizzas, hamburguesas y panchos tienen demasiada sal. Evita comerlos muy seguido.	11	8.25
		¿Sabias que papas fritas, chizitos y palitos pueden subirte la presion? Evita comerlos muy seguido.	12	8.00
		Lleva al trabajo comida preparada en casa, en vez de comprar sandwiches o comida rapida. La podes preparar con menos sal y sera mas sana.	8	7.75
		Proba preparar tu sandwich con tomate, lechuga y pollo en lugar de fiambres o embutidos, que tienen mucha sal.	10	7.75
	Maintenance	¿Sabias que al comer menos sal te estas ahorrando muchos gastos medicos en el futuro?	16	9.50
		Acordate que comiendo menos pizza, papas fritas, salchichas y hamburguesas proteges tu corazon.	15	9.00
		Continua protegiendo tu corazon. Consume menos de una cucharadita de sal al dia.	13	7.50
		Si continuas poniendo menos sal a las comidas ayudaras a que tu presion no suba.	14	7.50

ARGENTINA - SMS Appeal Ranking: Average scores by domain and stage of change (2/4)

Domain	Stage of change	Text Message (SMS)	SMS ID	Average score
Fruit and vegetable intake	Precontemplation & contemplation	Las frutas y verduras no tienen grasa, por eso te ayudan a bajar de peso y a verte bien.	17	9.50
		¿Sabias que las frutas y las verduras tienen vitaminas y muchas fibras? Ahora ya tenes otra razon para comerlas!	19	9.25
		Contales a las mujeres de tu casa que las frutas y las verduras ayudan a bajar el colesterol y protegen el corazon.	18	8.75
		Verduras como tomates, espinaca, brocolis, acelga y calabazas te ayudan a tener la presion normal.	21	8.25
		Lo ideal para estar sano es comer 5 frutas o verduras al dia. Intentalo!	22	8.25
		Frutas como bananas, ciruelas, melones, duraznos y naranjas te ayudan a tener la presion normal.	20	7.50
	Preparation & action	Cuida a tu familia dandole el ejemplo. Intenta comer frutas y verduras todos los dias.	26	9.75
		¿Por que no haces algo distinto? Empezar tu dia con una fruta te ayudara a estar sano y a no engordar.	25	9.50
		Las frutas de estacion no son caras y son sabrosas. Aprovechalas cuando vayas al mercado.	24	8.25
		Cuida tu corazon comiendo frutas y verduras en la merienda en lugar de facturas, bizcochos y galletitas.	23	8.00
		Para comer mas verduras podes agregarlas a tus sandwiches, arroz, carne y fideos.	28	7.75
		¿Ya comiste alguna fruta o verdura hoy? Trata de comer al menos una en cada comida.	27	7.75
	Maintenance	Continua comiendo al menos 5 frutas y verduras al dia. Protegen tu corazon.	32	9.75
		Al comer frutas y verduras todos los dias estas ayudando a tu salud. Recorda que tu salud lo vale!	29	8.75
		Continua comiendo frutas y verduras como banana, melon, ciruela, tomate, espinaca, brocoli y zapallo. Hacen bien!	30	7.25
		Sigue asi. Las frutas y verduras no tienen grasa, por eso te ayudan a bajar de peso y a verte bien.	31	7.00

ARGENTINA - SMS Appeal Ranking: Average scores by domain and stage of change (3/4)

Domain	Stage of change	Text Message (SMS)	SMS ID	Average score
Consumption of high-fat and sugar foods	Precontemplation & contemplation	¿Sabias que si en vez de freir la comida la haces al horno estas cuidando tu corazon y el de tu familia?	33	10.00
		¿Sabias que la margarina, las facturas, las galletitas, y las tortas te suben el colesterol y la presion?	37	9.50
		Si comes menos facturas,alfajores, chocolates, golosinas y galletitas, que tienen muchisima grasa y azucar, vas a bajar de peso.	35	9.00
		Evita comer hamburguesas, pizza y panchos. Son altisimos en grasa y tienen mucha sal. Cuida tu corazon.	34	8.00
		¿Sabias que al dejar de tomar gaseosas cuidas tu figura y te proteges de muchas enfermedades?	38	7.75
		Si comes menos manteca, achuras, salchichas, jamon y otros embutidos, podras bajar tu colesterol y tu presion.	36	7.00
	Preparation & action	Al cocinar, proba usar aceites vegetales en vez de margarina o manteca. Tienen grasas buenas.	39	9.50
		En la merienda podes cambiar las galletitas, las facturas y las tortas por frutas. Te haran sentir mas liviano.	40	8.25
		Reduci la cantidad de azucar que le agregas al cafe, al mate, al te y a los jugos.	42	8.00
		Para comer menos grasa quitale el pellejo al pollo y la grasa a la carne antes de cocinarla o comerla.	44	7.50
		Para no engordar, en lugar de gaseosas y jugos artificiales con azucar, toma agua.	43	7.25
		Cuando comas afuera pedi una porcion pequeña o comparti tu comida con alguien mas. Asi vas a bajar de peso y ahorrar dinero.	41	5.25
	Maintenance	La tentacion de comer alimentos con grasa y azucar puede ser muy fuerte, pero recorda que tu salud esta primero.	48	9.25
		Continua evitando las gaseosas y los jugos artificiales. Tu cuerpo te lo agradecera.	47	9.00
		Continua evitando alimentos con muchas grasas y azucar para proteger tu corazon. Vos podes!	45	8.75
		Que buena noticia que ya estas cuidando tu corazon de las comidas y las bebidas con azucar.	46	8.25

ARGENTINA - SMS Appeal Ranking: Average scores by domain and stage of change (4/4)

Domain	Stage of change	Text Message (SMS)	SMS ID	Average score
Physical activity	Precontemplation & contemplation	Caminar, correr, bailar y hacer deporte son actividades fisicas. Intenta hacerlas 30 minutos al dia.	49	9.75
		¿Estas estresado o preocupado? Haciendo actividad fisica 30 minutos al dia te vas a sentir mas relajado, tranquilo y hasta vas a dormir mejor.	51	9.75
		Haciendo actividad fisica al menos 30 minutos al dia vas a bajar tu presion y evitar enfermedades.	50	8.75
		¿Pensaste cuanto tiempo pasas mirando tele o frente a la computadora? Para ser fisicamente activo solo necesitas 30 minutos al dia de actividad fisica!	52	8.67
		Hacer una actividad fisica puede ser dificil al comienzo, pero cuando empieces te vas a sentir con mas energia. Intentalo.	54	8.50
		¿No tenes tiempo para hacer actividad fisica? Solo necesitas caminar o hacer deporte 30 minutos al dia!	53	8.50
	Preparation & action	Planifica hacer actividad fisica o el ejercicio que mas te guste. Te vas a divertir y ademas te va a ayudar a bajar tu presion.	58	10.00
		Recorda que lo ideal es hacer 30 minutos al dia de actividad fisica o ejercicio. Comenza con algo facil para vos y subi poco a poco la cantidad de tiempo.	57	9.00
		Camina a tu trabajo, hace las compras a pie, juga con los niños o saca a pasear al perro. Todo suma.	56	8.33
		En vez de ver tele, busca amigos o familiares que te acompañen a caminar o a hacer otra actividad fisica. Eso lo hara mas facil y entretenido.	59	8.25
		Si tienes que ir cerca de tu casa no uses transporte publico. Es mejor caminar! Asi ahorras dinero y haces actividad fisica.	60	7.50
		Empezar a hacer actividad fisica es una muy buena decision ¡animo!	55	7.50
	Maintenance	Seguir haciendo ejercicio te va a ayudar a no engordar y a verte mejor.	63	9.75
		Hacer actividad fisica ha sido una excelente decision. Segui adelante!	61	9.00
		Hacer actividad fisica demuestra lo importante que es para vos cuidar tu cuerpo y sentirte bien.	62	8.75
		Seguir haciendo actividad fisica te ayuda a mantener tu presion normal.	64	8.00
		**Total**		**8.39**

GUATEMALA - SMS Appeal Ranking: Average scores by domain and stage of change (1/4)

Domain	Stage of change	Text Message (SMS)	SMS ID	Average score
Salt and sodium intake	Precontemplation & contemplation	Si quitas el salero de la mesa ayudaras a tu familia a comer con menos sal.	6	10.00
		Si preparas alimentos con poca sal ayudaras a tu familia a tener la presion normal.	1	9.50
		Una persona solo necesita media cucharadita de sal al dia, pero la mayoria de gente come mas ¿Cuanto comes tu? Piensalo!	5	9.50
		Para mantener tu presion normal evita comer hamburguesas, pizza, hot dogs y chucherias. Tienen muchisima sal.	2	9.25
		Puedes tener tu presion normal si usas menos sal en tu comida. Protege tu corazon.	3	8.25
		Los cubos de consome y sopas instantaneas tienen mucha sal. Trata de evitarlos.	4	7.00
	Preparation & action	Lleva al trabajo comida preparada en casa, en vez de comida rapida o chucherias. La puedes preparar con menos sal y sera mas sana.	8	10.00
		¿Sabias que las papalinas y las bolsitas de ricitos pueden subirte la presion? Evita comerlos muy seguido.	12	10.00
		Reduce poco a poco la sal en las comidas que cocines. Asi tu y tu familia se iran acostumbrando.	7	10.00
		Pizzas, hamburguesas y hot dogs tienen demasiada sal. Evita comerlos muy seguido.	11	9.50
		Prueba preparar tu sandwich con verduras, pollo o frijoles en lugar de jamones o embutidos, que tienen mucha sal.	10	9.00
		Prueba cambiar la sal del salero por pimienta, oregano o ajo.	9	7.50
	Maintenance	Continua protegiendo tu corazon. Consume menos de una cucharadita de sal al dia.	13	10.00
		¿Sabias que al comer menos sal te estas ahorrando muchos gastos medicos en el futuro?	16	9.50
		Si continuas poniendo menos sal a las comidas ayudaras a que tu presion no suba.	14	9.00
		Acuerdate que comiendo menos pizza, papas fritas, salchichas y hamburguesas proteges tu corazon.	15	8.50

GUATEMALA - SMS Appeal Ranking: Average scores by domain and stage of change (2/4)

Domain	Stage of change	Text Message (SMS)	SMS ID	Average score
Fruit and vegetable intake	Precontemplation & contemplation	Cuentales a las mujeres de tu hogar que las frutas y las verduras ayudan a bajar el colesterol y protegen el corazon.	18	10.00
		¡Lo ideal para estar sano es comer 5 frutas o verduras al dia. Intentalo!	22	9.75
		Verduras como tomate, espinaca, brocoli, acelga y ayote te ayudan a tener la presion normal.	21	9.75
		Las frutas y verduras no tienen grasa, por lo que te ayudan a bajar de peso y a verte bien.	17	9.50
		Frutas como bananos, ciruelas, melones, melocotones y naranjas te ayudan a tener la presion normal.	20	8.75
		¿Sabias que las frutas y las verduras tienen vitaminas y mucha fibra? ¡Ahora ya tienes otra razon para comerlas!	19	8.50
	Preparation & action	Cuida tu corazon comiendo frutas y verduras en la refaccion en lugar de bolsitas de ricitos.	23	10.00
		Cuida a tu familia dandole el ejemplo. Intenta comer frutas y verduras todos los dias.	26	10.00
		Las frutas de estacion no son caras y saben mejor. Aprovechalas cuando vayas al mercado.	24	10.00
		¿Ya comiste alguna fruta o verdura hoy? Trata de comer al menos una en cada comida.	27	9.50
		¿Por que no haces algo distinto? Empezar tu dia con una fruta te ayudara a estar sano y a no engordar.	25	9.25
		Para comer mas verduras puedes agregarlas a tus frijoles, huevos, panes, arroz, carne y fideos.	28	7.00
	Maintenance	Al comer frutas y verduras todos los dias estas ayudando a tu salud. Recuerda que tu salud lo vale!	29	10.00
		Sigue asi. Las frutas y verduras no tienen grasa, por lo que te ayudan a bajar de peso y a verte bien.	31	9.75
		Continua comiendo al menos 5 frutas y verduras al dia. Protegen tu corazon.	32	9.75
		Continua comiendo frutas y verduras como banano, melon, ciruela, tomate, espinaca, brocoli y ayote. Hacen bien!	30	9.50

GUATEMALA - SMS Appeal Ranking: Average scores by domain and stage of change (3/4)

Domain	Stage of change	Text Message (SMS)	SMS ID	Average score
**Consumption of high-fat and sugar foods**	**Precontemplation & contemplation**	¿Sabias que si en vez de freir la comida la haces al horno estaras cuidando tu corazon y el de tu familia?	33	10.00
		Si comes menos pasteles, chocolates, dulces y galletas, que tienen muchisima grasa y azucar, vas a bajar de peso.	35	10.00
		¿Sabias que al dejar de tomar aguas gaseosas y refrescos azucarados cuidas tu figura y te proteges de muchas enfermedades?	38	9.00
		Evita comer hamburguesas, pizza y hot dogs. Son altisimos en grasa y tienen mucha sal. Cuida tu Corazon	34	9.00
		¿Sabias que la margarina, el pan dulce, las galletas, y los pasteles te suben el colesterol y la presion?	37	8.75
		Si comes menos manteca, visceras, salchichas, jamon y otros embutidos, podras bajar tu colesterol y tu presion.	36	8.75
	**Preparation & action**	Para no engordar, en lugar de aguas gaseosas, jugos artificiales o refrescos con azucar, toma agua.	43	10.00
		Para comer menos grasa quitale el pellejo al pollo y el gordo a la carne antes de cocinarla o comerla.	44	9.75
		Reduce la cantidad de azucar que le agregas al cafe, te, frescos y jugos.	42	9.50
		Al cocinar, prueba usar aceites vegetales en vez de margarina, mantequilla o manteca Tienen grasas buenas	39	9.50
		Cuando comas fuera de casa ordena una porcion pequeña o comparte tu comida con alguien mas. Asi bajaras de peso y ahorraras dinero.	41	9.25
		En la refaccion puedes cambiar las galletas, los pasteles y las chucherias por frutas. Te haran sentir mas liviano.	40	8.75
	**Maintenance**	Continua evitando alimentos con muchas grasas y azucar para proteger tu corazon. Tu puedes!	45	9.75
		La tentacion de comer alimentos con grasa y azucar puede ser muy fuerte, pero recuerda que tu salud esta primero.	48	9.50
		Que buena noticia que ya estas cuidando tu corazon de las comidas y refrescos azucarados.	46	9.25
		Continua evitando las aguas gaseosas, los jugos artificiales y los refrescos azucarados. Tu cuerpo te lo agradecera.	47	7.75

GUATEMALA - SMS Appeal Ranking: Average scores by domain and stage of change (4/4)

Domain	Stage of change	Text Message (SMS)	SMS ID	Average score
**Physical activity**	**Precontemplation & contemplation**	Haciendo actividad fisica al menos 30 minutos al dia bajaras tu presion y evitaras enfermedades.	50	10.00
		Hacer una actividad fisica puede ser dificil al comienzo, pero cuando empieces te sentiras con mas energia. Intentalo.	54	10.00
		¿Te sientes estresado o preocupado? Haciendo actividad fisica 30 minutos al dia te sentiras mas tranquilo y hasta dormiras mejor.	51	9.75
		¿Has pensado cuanto tiempo pasas viendo television o frente a la computadora? Para ser fisicamente activo solo necesitas 30 minutos al dia de actividad fisica!	52	9.50
		¿No tienes tiempo para hacer actividad fisica? Solo necesitas caminar o hacer deporte 30 minutos al dia!	53	9.50
		Caminar, correr, bailar y hacer deportes son actividades fisicas. Intenta hacerlas 30 minutos al dia.	49	9.00
	**Preparation & action**	Si tienes que ir cerca de tu casa no tomes transporte publico. Es mejor caminar! Asi ahorraras dinero y estaras haciendo actividad fisica.	60	10.00
		En vez de ver television, busca amigos o familiares que te acompañen a caminar o a hacer otra actividad fisica. Eso lo hara mas facil y entretenido.	59	10.00
		Empezar a hacer actividad fisica es una muy buena decision ¡animo!	55	9.75
		Camina a tu trabajo, haz las compras a pie, juega con los niños o saca a pasear al perro. Todo cuenta.	56	9.25
		Planifica hacer la actividad fisica o el ejercicio que mas te guste. Te divertiras y ademas te ayudara a bajar tu presion.	58	9.25
		Recuerda que lo ideal es hacer 30 minutos al dia de actividad fisica o ejercicio. Comienza con algo facil para ti y sube poco a poco la cantidad de tiempo.	57	9.00
	**Maintenance**	Seguir haciendo actividad fisica te ayudara a mantener tu presion normal.	64	10.00
		Hacer actividad fisica demuestra lo importante que es para ti cuidar tu cuerpo y sentirte bien.	62	10.00
		Seguir haciendo ejercicio te ayudara a no engordar y a verte mejor.	63	9.75
		Hacer actividad fisica ha sido una excelente decision. Sigue adelante!	61	8.75
		**Total**		**9.37**

PERU - SMS Appeal Ranking: Average scores by domain and stage of change (1/4)

Domain	Stage of change	Text Message (SMS)	SMS ID	Average score
Salt and sodium intake	Precontemplation & contemplation	Si preparas alimentos con poca sal ayudaras a tu familia a tener la presion normal.	1	9.50
		Los cubitos de sopa y las sopas instantaneas tienen mucha sal. Trata de evitarlos.	4	8.50
		Para mantener tu presion normal evita comer hamburguesas, pizza, salchichas y productos como papitas o chizitos. Tienen muchisima sal.	2	8.25
		Si quitas el salero de la mesa ayudaras a tu familia a comer con menos sal.	6	8.25
		Una persona solo necesita media cucharadita de sal al dia, pero la mayoria de gente come mas ¿Cuanto comes tu? Piensalo!	5	8.00
		Puedes tener tu presion normal si usas menos sal en tu comida. Protege tu corazon.	3	8.00
	Preparation & action	Reduce poco a poco la sal en las comidas que cocines. Asi tu y tu familia se iran acostumbrando.	7	9.75
		Pizzas, hamburguesas y salchichas tienen demasiada sal. Evita comerlos muy seguido.	11	8.25
		¿Sabias que productos como las papitas y los chizitos pueden subirte la presion? Evita comerlos muy seguido.	12	8.25
		Lleva al trabajo comida preparada en casa, en vez de comprar sanguches o comida rapida. La puedes preparar con menos sal y sera mas sana.	8	8.00
		Prueba cambiar la sal del salero por pimienta, oregano o aji.	9	6.50
		Prueba preparar tu sanguche con tomate, lechuga y pollo en lugar de usar embutidos como jamon o salchichas, que tienen mucha sal.	10	6.00
	Maintenance	Si continuas poniendo menos sal a las comidas ayudaras a que tu presion no suba.	14	9.75
		Acuerdate que comiendo menos pizza, papas fritas, salchichas y hamburguesas proteges tu corazon.	15	9.00
		¿Sabias que al comer menos sal te estas ahorrando muchos gastos medicos en el futuro?	16	8.50
		Continua protegiendo tu corazon. Consume menos de una cucharadita de sal al dia.	13	8.25

PERU - SMS Appeal Ranking: Average scores by domain and stage of change (2/4)

Domain	Stage of change	Text Message (SMS)	SMS ID	Average score
Fruit and vegetable intake	Precontemplation & contemplation	Frutas como platanos, ciruelas, melones, melocotones y naranjas te ayudan a tener la presion normal.	20	9.75
		Las frutas y verduras no tienen grasa, por eso te ayudan a bajar de peso y a verte bien.	17	9.50
		Verduras como tomates, espinaca, brocolis, acelga y zapallo te ayudan a tener la presion normal.	21	8.75
		¡Lo ideal para estar sano es comer 5 frutas o verduras al dia. Intentalo!	22	8.50
		Cuentale a las mujeres de tu casa que las frutas y las verduras ayudan a bajar el colesterol y protegen el corazon.	18	8.25
		¿Sabias que las frutas y las verduras tienen vitaminas y muchas fibras? ¡Ahora ya tienes otra razon para comerlas!	19	7.75
	Preparation & action	Cuida a tu familia dandole el ejemplo. Intenta comer frutas y verduras todos los dias.	26	9.50
		¿Por que no haces algo distinto? Empezar tu dia con una fruta te ayudara a estar sano y a no engordar.	25	9.50
		¿Ya comiste alguna fruta o verdura hoy? Trata de comer al menos una en cada comida.	27	8.25
		Cuida tu corazon comiendo frutas y verduras como refrigerio, en lugar de papitas, galletas, dulces o sanguches.	23	8.25
		Para comer mas verduras puedes agregarlas a tus guisos, menestras, arroz, tallarines o sanguches.	28	8.00
		Las frutas de estacion no son caras y saben mejor. Aprovechalas cuando vayas al mercado.	24	7.00
	Maintenance	Sigue asi. Las frutas y verduras no tienen grasa, por lo que te ayudan a bajar de peso y a verte bien.	31	9.25
		Continua comiendo frutas y verduras como platano, melon, ciruela, tomate, espinaca, brocoli y zapallo. Hacen bien!	30	9.25
		Al comer frutas y verduras todos los dias estas ayudando a tu salud. Recuerda que tu salud lo vale!	29	9.00
		Continua comiendo al menos 5 frutas y verduras al dia. Protegen tu corazon.	32	7.75

PERU - SMS Appeal Ranking: Average scores by domain and stage of change (3/4)

Domain	Stage of change	Text Message (SMS)	SMS ID	Average score
Consumption of high-fat and sugar foods	Precontemplation & contemplation	¿Sabias que la margarina, las tortas, los pasteles y las galletas te suben el colesterol y la presion?	37	9.50
		¿Sabias que si en vez de freir la comida la haces al horno estaras cuidando tu corazon y el de tu familia?	33	8.75
		¿Sabias que al dejar de tomar gaseosas y refrescos con azucar cuidas tu figura y te proteges de muchas enfermedades?	38	8.75
		Evita comer hamburguesas, pizza, pollo frito y salchichas. Son altisimos en grasa y tienen mucha sal. Cuida tu Corazon	34	8.25
		Si comes menos tortas, pasteles, chocolates, golosinas y galletas, que tienen muchisima grasa y azucar, vas a bajar de peso.	35	7.50
		Si comes menos manteca, visceras, salchichas, jamon y otros embutidos, podras bajar tu colesterol y tu presion.	36	5.00
	Preparation & action	Para comer menos grasa quitale el pellejo al pollo y la grasa a la carne antes de cocinarla o comerla.	44	9.25
		Para no engordar, en lugar de gaseosas, jugos en caja o refrescos con azucar, toma agua.	43	9.25
		Al cocinar, prueba usar aceites vegetales en vez de margarina, mantequilla o manteca Tienen grasas buenas	39	8.75
		En tu refrigerio puedes cambiar las galletas, los pasteles o las papitas por frutas. Te haran sentir mas liviano.	40	8.25
		Reduce la cantidad de azucar que le agregas al cafe, te, refrescos y jugos.	42	6.00
		Cuando comas fuera de casa compra una porcion pequeña o comparte tu comida con alguien mas. Asi bajaras de peso y ahorraras dinero.	41	4.50
	Maintenance	Continua evitando las gaseosas, los jugos en caja y los refrescos con azucar. Tu cuerpo te lo agradecera.	47	9.75
		Continua evitando alimentos con muchas grasas y azucar para proteger tu corazon. Tu puedes!	45	9.00
		La tentacion de comer alimentos con grasa y azucar puede ser muy fuerte, pero recuerda que tu salud esta primero.	48	6.75
		Que buena noticia que ya estas cuidando tu corazon de las comidas y bebidas con azucar.	46	5.50

PERU - SMS Appeal Ranking: Average scores by domain and stage of change (4/4)

Domain	Stage of change	Text Message (SMS)	SMS ID	Average score
Physical activity	Precontemplation & contemplation	¿Te sientes preocupado o estresado? Haciendo actividad fisica 30 minutos al dia te sentiras mas relajado, tranquilo y hasta dormiras mejor.	51	9.50
		¿No tienes tiempo para hacer actividad fisica? Solo necesitas caminar o hacer deporte 30 minutos al dia!	53	8.75
		Haciendo actividad fisica al menos 30 minutos al dia podras bajar tu presion y evitar enfermedades.	50	8.00
		Hacer una actividad fisica puede ser dificil al comienzo, pero cuando empieces te sentiras con mas energia. Intentalo.	54	8.00
		Caminar, correr, bailar y hacer deporte son actividades fisicas. Intenta hacerlas 30 minutos al dia.	49	7.00
		¿Has pensado cuanto tiempo pasas viendo television o en la computadora? Para ser fisicamente activo solo necesitas 30 minutos al dia de actividad fisica!	52	5.75
	Preparation & action	Empezar a hacer actividad fisica es una muy buena decision ¡animo!	55	9.50
		Si tienes que ir cerca de tu casa no tomes combi o mototaxi. Es mejor caminar! Asi ahorras dinero y haces actividad fisica.	60	9.25
		Recuerda que lo ideal es hacer 30 minutos al dia de actividad fisica o ejercicio. Comienza con algo facil para ti y sube poco a poco la cantidad de tiempo.	57	9.25
		En vez de ver television, busca amigos o familiares que te acompañen a caminar o a hacer otra actividad fisica. Eso lo hara mas facil y entretenido.	59	8.50
		Planifica hacer la actividad fisica o el ejercicio que mas te guste. Te divertiras y ademas te ayudara a bajar tu presion.	58	8.25
		Camina a tu trabajo, anda a hacer las compras a pie, juega con los niños o saca a pasear al perro. Todo cuenta	56	7.75
	Maintenance	Seguir haciendo ejercicio te ayudara a no engordar y a verte mejor.	63	9.75
		Hacer actividad fisica ha sido una excelente decision. Sigue adelante!	61	9.50
		Seguir haciendo actividad fisica te ayudara a mantener tu presion normal.	64	9.25
		Hacer actividad fisica demuestra lo importante que es para ti cuidar tu cuerpo y sentirte bien.	62	9.00
		**Total**		**8.32**
