# Supplementary material for: Design and Multi-Country Validation of Text Messages for an mHealth Intervention for Primary Prevention of Progression to Hypertension in Latin America
Source: JMIR Mhealth Uhealth. 2015 Feb 18;3(1):e19. doi: 10.2196/mhealth.3874 (PMC4376187; doi:10.2196/mhealth.3874)
Supplement: Supplementary file 3 [file mhealth_v3i1e19_app3.pdf]

ARGENTINA –Final list of SMS (1/4)

Domain	Stage of change	Text Message (SMS)	SMS ID
Salt and sodium intake	Precontemplation & contemplation	Si preparas alimentos con poca sal ayudaras a tu familia a tener la presion normal.	1
		Podes tener tu presion normal si usas menos sal en tu comida. Protege tu corazon.	3
		Para mantener tu presion normal evita comer hamburguesas, pizza, panchos, papas fritas y manies. Tienen muchisima sal.	2
		Una persona solo necesita media cucharadita de sal al dia, ¿Cuanto comes tu? Pensalo!	5
		Si sacas el salero de la mesa ayudaras a tu familia a comer con menos sal.	6
	Preparation & action	Reduce poco a poco la sal en las comidas que cocines. Asi vos y tu familia se iran acostumbrando.	7
		Proba cambiar la sal del salero por pimienta, oregano o aji.	9
		Pizzas, hamburguesas y panchos tienen demasiada sal. Evita comerlos muy seguido.	11
		¿Sabias que papas fritas, chizitos y palitos pueden subirte la presion? Evita comerlos muy seguido.	12
		Intenta llevar al trabajo comida preparada en casa, en vez de comprar sandwiches o comida rapida. Peparala con menos sal y sera mas sana.	8
	Maintenance	¿Sabias que al comer menos sal te estas ahorrando muchos gastos medicos en el futuro?	16
		Acordate que comiendo menos pizza, papas fritas, salchichas y hamburguesas proteges tu corazon.	15
		Continua protegiendo tu corazon. Consume menos de una cucharadita de sal al dia.	13
		Si continuas poniendo menos sal a las comidas ayudaras a que tu presion no suba.	14

ARGENTINA –Final list of SMS (2/4)

Domain	Stage of change	Text Message (SMS)	SMS ID
**Fruit and vegetable intake**	**Precontemplation & contemplation**	Las frutas y verduras no tienen grasa, por eso te ayudan a bajar de peso y a verte bien.	17
		¿Sabias que las frutas y las verduras tienen vitaminas y muchas fibras? Ahora ya tenes otra razon para comerlas!	19
		Contales a todos en tu casa que las frutas y las verduras ayudan a bajar el colesterol y protegen el corazon.	18
		Verduras como tomates, espinaca, brocolis, acelga y calabazas te ayudan a tener la presion normal.	21
		Lo ideal para estar sano es comer 5 frutas o verduras al dia. Intentalo!	22
	**Preparation & action**	Cuida a tu familia dandole el ejemplo. Intenta comer frutas y verduras todos los dias.	26
		¿Por que no haces algo distinto? Empezar tu dia con una fruta te ayudara a estar sano y a no subir de peso.	25
		Las frutas de estacion son más baratas y son sabrosas. Aprovechalas cuando vayas al mercado.	24
		Cuida tu corazon comiendo frutas y verduras en la merienda en lugar de facturas, bizcochos y galletitas.	23
		¿Ya comiste alguna fruta o verdura hoy? Trata de comer al menos una en cada comida.	27
	**Maintenance**	Continua comiendo al menos 5 frutas y verduras al dia. Protegen tu corazon.	32
		Al comer frutas y verduras todos los dias estas ayudando a tu salud. Recorda que tu salud lo vale!	29
		Continua comiendo tus frutas o verduras favoritas. Hacen bien!	30
		Sigue asi. Las frutas y verduras no tienen grasa, por eso te ayudan a bajar de peso y a sentirte bien.	31

ARGENTINA –Final list of SMS (3/4)

Domain	Stage of change	Text Message (SMS)	SMS ID
**Consumption of high-fat and sugar foods**	**Precontemplation & contemplation**	¿Sabias que si en vez de freir la comida la haces al horno estas cuidando tu corazon y el de tu familia?	33
		¿Sabias que la margarina, las facturas, las galletitas, y las tortas te suben el colesterol y la presion?	37
		Si comes menos facturas,alfajores, chocolates, golosinas y galletitas, que tienen muchisima grasa y azucar, vas a bajar de peso.	35
		Evita comer hamburguesas, pizza y hot dogs. Son altisimos en grasa y tienen mucha sal. Cuida tu corazon.	34
		¿Sabias que al dejar de tomar gaseosas cuidas tu figura y te proteges de muchas enfermedades?	38
	**Preparation & action**	Al cocinar, proba usar aceites vegetales en vez de margarina o manteca. Tienen grasas buenas.	39
		En la merienda podes cambiar las galletitas, las facturas y las tortas por frutas. Te haran sentir mas liviano.	40
		Intenta reducir la cantidad de azucar que le agregas al cafe, al mate, al te y a los jugos.	42
		Para comer menos grasa quitale el pellejo al pollo y la grasa a la carne antes de cocinarla o comerla.	44
		Para no engordar, en lugar de gaseosas y jugos artificiales con azucar, toma agua.	43
	**Maintenance**	La tentacion de comer alimentos con grasa y azucar puede ser muy fuerte, pero recorda que tu salud esta primero.	48
		Continua evitando las gaseosas y los jugos artificiales. Tu cuerpo te lo agradecera.	47
		Continua evitando alimentos con muchas grasas y azucar para proteger tu corazon. Vos podes!	45
		Que buena noticia que ya estas cuidando tu corazon de las comidas y las bebidas con azucar.	46

ARGENTINA –Final list of SMS (4/4)

Domain	Stage of change	Text Message (SMS)	SMS ID
**Physical activity**	**Precontemplation & contemplation**	Caminar, correr, bailar y hacer deportes son actividades fisicas. Intenta hacerlas 30 minutos al dia.	49
		¿Estas estresado o preocupado? Haciendo actividad fisica 30 minutos al dia te vas a sentir mas relajado, tranquilo y hasta vas a dormir mejor.	51
		Haciendo actividad fisica al menos 30 minutos al dia vas a bajar tu presion y evitar enfermedades.	50
		¿Pensaste cuanto tiempo pasas mirando tele o frente a la computadora? Para ser una persona activa solo necesitas 30 minutos al dia de actividad fisica!	52
		Hacer una actividad fisica puede ser dificil al comienzo, pero cuando empieces te vas a sentir con mas energia. Intentalo.	54
	**Preparation & action**	Planifica hacer actividad fisica o el ejercicio que mas te guste. Te vas a divertir y ademas te va a ayudar a bajar tu presion.	58
		Recorda que lo ideal es hacer 30 minutos al dia de actividad fisica o ejercicio. Comenza con algo facil para vos y subi poco a poco la cantidad de tiempo.	57
		Camina a tu trabajo, hace las compras a pie, juga con los niños o saca a pasear al perro. Todo suma.	56
		En vez de ver tele, busca amigos o familiares que te acompañen a caminar o a hacer otra actividad fisica. Eso lo hara mas facil y entretenido.	59
		Empezar a hacer actividad fisica es una muy buena decision ¡animo!	55
	**Maintenance**	Seguir haciendo ejercicio te va a ayudar a no engordar y a sentirte bien.	63
		Hacer actividad fisica ha sido una excelente decision. Segui adelante!	61
		Hacer actividad fisica demuestra lo importante que es para vos cuidar tu cuerpo y sentirte bien.	62
		Seguir haciendo actividad fisica te ayuda a mantener tu presion normal.	64

GUATEMALA –Final list of SMS (1/4)

Domain	Stage of change	Text Message (SMS)	SMS ID
**Salt and sodium intake**	**Precontemplation & contemplation**	Si quitas el salero de la mesa ayudaras a tu familia a comer con menos sal.	6
		Si preparas alimentos con poca sal ayudaras a tu familia a tener la presion normal.	1
		Una persona solo necesita media cucharadita de sal al dia. ¿Cuanto comes tu? Piensalo!	5
		Para mantener tu presion normal evita comer hamburguesas, pizza, hot dogs y chucherias. Tienen muchisima sal.	2
		Puedes tener tu presion normal si usas menos sal en tu comida. Protege tu corazon.	3
	**Preparation & action**	Lleva al trabajo comida preparada en casa, en vez de comida rapida o chucherias. La puedes preparar con menos sal y sera mas sana.	8
		¿Sabias que las papalinas y las bolsitas de ricitos pueden subirte la presion? Evita comerlos muy seguido.	12
		Reduce poco a poco la sal en las comidas que cocines. Asi tu y tu familia se iran acostumbrando.	7
		Pizzas, hamburguesas y hot dogs tienen demasiada sal. Evita comerlos muy seguido.	11
		Prueba preparar tu sandwich con verduras, pollo o frijoles en lugar de jamones o embutidos, que tienen mucha sal.	10
	**Maintenance**	Continua protegiendo tu corazon. Consume menos de una cucharadita de sal al dia.	13
		¿Sabias que al comer menos sal te estas ahorrando muchos gastos medicos en el futuro?	16
		Si continuas poniendo menos sal a las comidas ayudaras a que tu presion no suba.	14
		Acuerdate que comiendo menos pizza, papas fritas, salchichas y hamburguesas proteges tu corazon.	15

GUATEMALA –Final list of SMS (2/4)

Domain	Stage of change	Text Message (SMS)	SMS ID
Fruit and vegetable intake	Precontemplation & contemplation	Cuentale a todos en tu hogar que las frutas y las verduras ayudan a bajar el colesterol y protegen el corazon.	18
		¡Lo ideal para estar sano es comer 5 frutas o verduras al dia. Intentalo!	22
		Verduras como tomate, espinaca, brocoli, acelga y ayote te ayudan a tener la presion normal.	21
		Las frutas y verduras no tienen grasa, por lo que te ayudan a bajar de peso y a verte bien.	17
		Frutas como bananos, ciruelas, melones, melocotones y naranjas te ayudan a tener la presion normal.	20
	Preparation & action	Cuida tu corazon comiendo frutas y verduras en la refaccion en lugar de bolsitas de ricitos.	23
		Cuida a tu familia dandole el ejemplo. Intenta comer frutas y verduras todos los dias.	26
		Las frutas de estacion no son caras y saben mejor. Aprovechalas cuando vayas al mercado.	24
		¿Ya comiste alguna fruta o verdura hoy? Trata de comer al menos una en cada comida.	27
		¿Por que no haces algo distinto? Empezar tu dia con una fruta te ayudara a estar sano y a no subir de peso.	25
	Maintenance	Al comer frutas y verduras todos los dias estas ayudando a tu salud. Recuerda que tu salud lo vale!	29
		Sigue asi. Las frutas y verduras no tienen grasa, por lo que te ayudan a bajar de peso y a verte bien.	31
		Continua comiendo al menos 5 frutas y verduras al dia. Protegen tu corazon.	32
		Continua comiendo tus frutas o verduras favoritas. Hacen bien!	30

GUATEMALA –Final list of SMS (3/4)

Domain	Stage of change	Text Message (SMS)	SMS ID
**Consumption of high-fat and sugar foods**	**Precontemplation & contemplation**	¿Sabias que si en vez de freir la comida la haces al horno estaras cuidando tu corazon y el de tu familia?	33
		Si comes menos pasteles, chocolates, dulces y galletas, que tienen muchisima grasa y azucar, vas a bajar de peso.	35
		¿Sabias que al dejar de tomar aguas gaseosas y refrescos azucarados cuidas tu figura y te proteges de muchas enfermedades?	38
		Evita comer hamburguesas, pizza y hot dogs. Son altisimos en grasa y tienen mucha sal. Cuida tu Corazon	34
		Si comes menos manteca, visceras, salchichas, jamon y otros embutidos, podras bajar tu colesterol y tu presion.	36
	**Preparation & action**	Para no engordar, en lugar de aguas gaseosas, jugos artificiales o refrescos con azucar, toma agua.	43
		Para comer menos grasa quitale el pellejo al pollo y el gordo a la carne antes de cocinarla o comerla.	44
		Reduce la cantidad de azucar que le agregas al cafe, te, frescos y jugos. Asi tendras un buen peso.	42
		Al cocinar, prueba usar aceites vegetales en vez de margarina, mantequilla o manteca Tienen grasas buenas	39
		En la refaccion come frutas en lugar de galletas y chucherias. La fruta te hara sentir mas liviano.	40
	**Maintenance**	Continua evitando alimentos con muchas grasas y azucar para proteger tu corazon. Tu puedes!	45
		A veces tendras muchas ganas de comer alimentos con grasa y azucar, pero recuerda que tu salud esta primero.	48
		Que buena noticia que ya estas cuidando tu corazon de las comidas y refrescos azucarados.	46
		Continua evitando las aguas gaseosas, los jugos artificiales y los refrescos azucarados. Tu cuerpo te lo agradecera.	47

GUATEMALA –Final list of SMS (4/4)

Domain	Stage of change	Text Message (SMS)	SMS ID
**Physical activity**	**Precontemplation & contemplation**	Haciendo actividad fisica al menos 30 minutos al dia bajaras tu presion y evitaras enfermedades.	50
		Hacer una actividad fisica puede ser dificil al comienzo, pero cuando empieces te sentiras con mas energia. Intentalo.	54
		¿Te sientes estresado o preocupado? Haciendo actividad fisica 30 minutos al dia te sentiras mas tranquilo y hasta dormiras mejor.	51
		¿Has pensado cuanto tiempo pasas viendo television o frente a la computadora? Para ser una persona activa solo necesitas 30 minutos al dia de actividad fisica!	52
		Caminar, correr, bailar y hacer deportes son actividades fisicas. Intenta hacerlas 30 minutos al dia.	49
	**Preparation & action**	Si tienes que ir cerca de tu casa no tomes transporte publico. Es mejor caminar! Asi ahorraras dinero y estaras haciendo actividad fisica.	60
		En vez de ver television, busca amigos o familiares que te acompañen a caminar o a hacer otra actividad fisica. Eso lo hara mas facil y entretenido.	59
		Empezar a hacer actividad fisica es una muy buena decision ¡animo!	55
		Camina a tu trabajo, haz las compras a pie, juega con los niños o saca a pasear al perro. Todo cuenta.	56
		Recuerda que lo ideal es hacer 30 minutos al dia de actividad fisica o ejercicio. Comienza con algo facil para ti y sube poco a poco la cantidad de tiempo.	57
	**Maintenance**	Seguir haciendo actividad fisica te ayudara a mantener tu presion normal.	64
		Hacer actividad fisica demuestra lo importante que es para ti cuidar tu cuerpo y sentirte bien.	62
		Seguir haciendo ejercicio te ayudara a no engordar y a verte mejor.	63
		Hacer actividad fisica ha sido una excelente decision. Sigue adelante!	61

PERU –Final list of SMS (1/4)

Domain	Stage of change	Text Message (SMS)	SMS ID
**Salt and sodium intake**	**Precontemplation & contemplation**	Si preparas alimentos con poca sal ayudaras a tu familia a tener la presion normal.	1
		Los cubitos de caldo y las sopas instantaneas tienen mucha sal. Trata de evitarlos.	4
		Para mantener tu presion normal evita comer hamburguesas, pizza, salchichas y productos como papitas o chizitos. Tienen muchisima sal.	2
		Si quitas el salero de la mesa ayudaras a tu familia a comer con menos sal.	6
		Puedes tener tu presion normal si usas menos sal en tu comida. Protege tu corazon.	3
	**Preparation & action**	Reduce poco a poco la sal en las comidas que cocines. Asi tu y tu familia se iran acostumbrando.	7
		Pizzas, hamburguesas y embutidos tienen demasiada sal. Evita comerlos muy seguido.	11
		¿Sabias que productos como las papitas y los chizitos pueden subirte la presion? Evita comerlos muy seguido.	12
		Lleva al trabajo comida preparada en casa. Puedes pepararla con menos sal y sera mas sana que la comida de la calle.	8
		Los embutidos como jamon, jamonada y chorizo tienen mucha sal. Mejor preparara tu sanguche con pollo, tomate y lechuga.	10
	**Maintenance**	Si continuas poniendo menos sal a las comidas ayudaras a que tu presion no suba.	14
		Acuerdate que comiendo menos pizza, papas fritas, salchichas y hamburguesas proteges tu corazon.	15
		¿Sabias que al comer menos sal te estas ahorrando muchos gastos medicos en el futuro?	16
		Continua protegiendo tu corazon. Consume menos de una cucharadita de sal al dia.	13

PERU –Final list of SMS (2/4)

Domain	Stage of change	Text Message (SMS)	SMS ID
Fruit and vegetable intake	Precontemplation & contemplation	Frutas como platanos, ciruelas, melones, melocotones y naranjas te ayudan a tener la presion normal.	20
		Las frutas y verduras no tienen grasa, por eso te ayudan a bajar de peso y a verte bien.	17
		Verduras como tomates, espinaca, brocolis, acelga y zapallo te ayudan a tener la presion normal.	21
		¡Lo ideal para estar sano es comer 5 frutas o verduras al dia. Intentalo!	22
		Cuentale a todos en tu casa que las frutas y las verduras ayudan a bajar el colesterol y protegen el corazon.	18
	Preparation & action	Cuida a tu familia dandole el ejemplo. Intenta comer frutas y verduras todos los dias.	26
		Empezar tu dia con una fruta o un jugo natural te ayudara a estar sano y a no engordar.	25
		¿Ya comiste alguna fruta o verdura hoy? Trata de comer al menos una en cada comida.	27
		Cuida tu corazon. Come frutas y verduras como refrigerio, en vez de papitas o dulces.	23
		Las frutas de estacion son más baratas y saben mejor. Aprovechalas cuando vayas al mercado.	24
	Maintenance	Sigue asi. Las frutas y verduras no tienen grasa, por lo que te ayudan a bajar de peso y a verte bien.	31
		Continua comiendo tus frutas o verduras favoritas. Hacen bien!	30
		Al comer frutas y verduras todos los dias estas ayudando a tu salud. Recuerda que tu salud es lo primero!	29
		Continua comiendo al menos 5 frutas y verduras al dia. Protegen tu corazon.	32

PERU –Final list of SMS (3/4)

Domain	Stage of change	Text Message (SMS)	SMS ID
**Consumption of high-fat and sugar foods**	**Precontemplation & contemplation**	¿Sabias que la margarina, las tortas, los pasteles y las galletas te suben el colesterol y la presion?	37
		¿Sabias que si en vez de freir la comida la haces al horno estaras cuidando tu corazon y el de tu familia?	33
		¿Sabias que al dejar de tomar gaseosas y refrescos con azucar cuidas tu figura y te proteges de muchas enfermedades?	38
		Evita comer hamburguesas, pizza, pollo frito y salchichas. Son altisimos en grasa y tienen mucha sal. Cuida tu Corazon	34
		Si comes menos tortas, pasteles, chocolates, golosinas y galletas, que tienen muchisima grasa y azucar, vas a bajar de peso.	35
	**Preparation & action**	Para comer menos grasa quitale el pellejo al pollo y la grasa a la carne antes de cocinarla o comerla.	44
		Para no engordar, en lugar de gaseosas, jugos en caja o refrescos con azucar, toma agua.	43
		El aceite vegetal tiene grasas buenas para tu salud. Usalo al cocinar en vez de usar margarina, mantequilla o manteca.	39
		En tu refrigerio come fruta en lugar de galletas o papitas. Te sentiras mas liviano.	40
		Reduce la cantidad de azucar que le agregas al cafe, te, refrescos y jugos. Asi tendras un buen peso.	42
	**Maintenance**	Continua evitando las gaseosas, los jugos en caja y los refrescos con azucar. Tu cuerpo te lo agradecera.	47
		Continua evitando alimentos con muchas grasas y azucar para proteger tu corazon. Tu puedes!	45
		A veces tendras muchas ganas de comer alimentos con grasa y azucar, pero recuerda que tu salud esta primero.	48
		Que bueno que comes menos dulces y bebidas con azucar. Asi cuidas tu corazon.	46

PERU –Final list of SMS (4/4)

Domain	Stage of change	Text Message (SMS)	SMS ID
Physical activity	Precontemplation & contemplation	¿Te sientes preocupado o estresado? Haciendo actividad fisica 30 minutos al dia te sentiras mas relajado, tranquilo y hasta dormiras mejor.	51
		¿No tienes tiempo para hacer actividad fisica? Solo necesitas caminar o hacer deporte 30 minutos al dia!	53
		Haciendo actividad fisica al menos 30 minutos al dia podras bajar tu presion y evitar enfermedades.	50
		Hacer una actividad fisica puede ser dificil al comienzo, pero cuando empieces te sentiras con mas energia. Intentalo.	54
		Caminar, correr, bailar y hacer deporte son actividades fisicas. Intenta hacerlas 30 minutos al dia.	49
	Preparation & action	Empezar a hacer actividad fisica es una muy buena decision ¡animo!	55
		Si tienes que ir cerca de tu casa no tomes combi o mototaxi. Es mejor caminar! Asi no gastas en pasaje y haces actividad fisica.	60
		Recuerda que lo ideal es hacer 30 minutos al dia de actividad fisica o ejercicio. Comienza con algo facil para ti y sube poco a poco la cantidad de tiempo.	57
		En vez de ver television, busca amigos o familiares que te acompañen a caminar o a hacer otra actividad fisica. Eso lo hara mas facil y entretenido.	59
		Planifica hacer la actividad fisica o el ejercicio que mas te guste. Te divertiras y ademas te ayudara a bajar tu presion.	58
	Maintenance	Seguir haciendo ejercicio te ayudara a no engordar y a verte mejor.	63
		Hacer actividad fisica ha sido una excelente decision. Sigue adelante!	61
		Seguir haciendo actividad fisica te ayudara a mantener tu presion normal.	64
		Hacer actividad fisica demuestra lo importante que es para ti cuidar tu cuerpo y sentirte bien.	62
